# Education and training programmes for infection prevention and control professionals: mapping the current opportunities and local needs in European countries

**DOI:** 10.1186/s13756-020-00835-1

**Published:** 2020-11-09

**Authors:** Constantinos Tsioutis, Gabriel Birgand, Erik Bathoorn, Aleksander Deptula, Lenny ten Horn, Enrique Castro-Sánchez, Oana Săndulescu, Andreas F. Widmer, Athanasios Tsakris, Giulio Pieve, Evelina Tacconelli, Nico T. Mutters

**Affiliations:** 1 School of Medicine, European University Cyprus, Nicosia, Cyprus; 2 European Committee On Infection Control (EUCIC), Basel, Switzerland; 3 NIHR Health Protection Research Unit in Healthcare Associated Infection and Antimicrobial Resistance, Imperial College London, Hammersmith Campus, Du Cane Road, London, UK; 4 Department of Medical Microbiology, University of Groningen, University Medical Center Groningen, Groningen, The Netherlands; 5 Department of Propaedeutics of Medicine and Infection Prevention, Ludwik Rydygier Collegium Medicum in Bydgoszcz, Nicolaus Copernicus University in Toruń, Antimicrobial Stewardship and Infection Control Unit, Antoni Jurasz University Hospital No. 1, Bydgoszcz, Poland; 6 Imperial College Healthcare NHS Trust, St Mary’s Road, London, UK; 7 Carol Davila University of Medicine and Pharmacy, Bucharest, Romania; 8 National Institute for Infectious Diseases “Prof. Dr. Matei Balș”, No. 1 Dr. Calistrat Grozovici Street, 021105 Bucharest, Romania; 9 Infectious Diseases and Hospital Epidemiology, University Hospital Basel, Petersgraben 4, 4031 Basel, Switzerland; 10 Department of Microbiology, Medical School, University of Athens, 11527 Athens, Greece; 11 Clinical management staff, University Hospital of Pisa, Pisa, Italy; 12 Infectious Diseases, Department of Diagnostic and Public Health, Verona University Hospital, Verona, Italy; 13 Institute for Hygiene and Public Health, University Hospital Bonn, Bonn, Germany

**Keywords:** Infection prevention, Infection control, Training, Education, Certification, Qualifications

## Abstract

**Background:**

Studies have repeatedly highlighted the need for homogenisation of training content and opportunities in infection prevention and control (IPC) across European countries.

**Objectives:**

To map current training opportunities for IPC professionals, define local needs and highlight differences, across 11 European countries (Cyprus, France, England, Germany, Greece, Italy, Netherlands, Poland, Romania, Spain, Switzerland).

**Sources:**

From July 2018 to February 2019, IPC experts directly involved in IPC training and education in their countries and/or internationally were invited to complete a prespecified set of questions in order to provide a detailed description of IPC training opportunities and needs in their country.

**Conclusions:**

IPC training among nurses and doctors varies greatly across countries, with differences in content and type of training (e.g., standardised curriculum, educational programme, clinical experience) duration, as well as in assessment and recognition/accreditation. The observed heterogeneity in IPC training between European countries can be eliminated through establishment of interdisciplinary region-wide training programmes, with common learning objectives, shared know-how and supported by national and international professional bodies.

## Background

The latest World Health Organization strategic plan emphasised ten global health threats. Among them, five were directly related to infection prevention and control (IPC) [1]. To tackle these threats in healthcare settings, and ensure accurate and sustainable implementation of best practices, hospital IPC teams need to be adequately staffed and include appropriately trained and educated members. In Europe, several organisations and working groups have provided oversight and direction, including: the European Center for Disease Prevention and Control (ECDC)-commissioned ‘Training in Infection Control in Europe’ (TRICE) project [2, 3], the Prevention of Hospital Infections by Intervention and Training (PROHIBIT) study [4] and the European Committee on Infection Control (EUCIC) [5]. The heterogeneity of IPC training content and opportunities across European countries has been repeatedly highlighted in studies [2, 4]. This observed heterogeneity hampers the implementation of national and international containment strategies, while limiting the capability to share IPC expertise and know-how between countries.

Several European initiatives were launched to bridge the gap in education and training. In 2013, the ECDC issued a comprehensive list of core competencies for IPC professionals, to be used in assessments and curricula, to enhance and homogenise the IPC capacities across European countries [3]. More recently, in 2015, the European Society of Clinical Microbiology and Infectious Diseases (ESCMID) launched the EUCIC. This standing committee aims to develop and establish a European-wide IPC training programme [5]. However, these initiatives need to be further supported by a detailed assessment of the current educational opportunities and local needs across European countries.

**Fig. 1:**
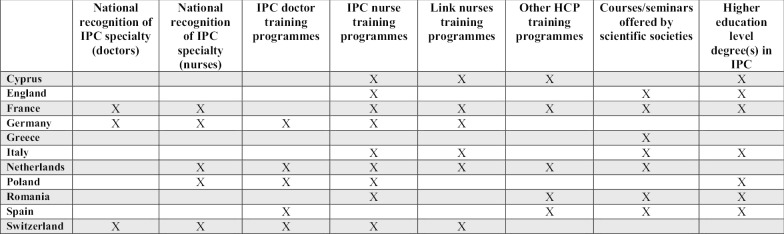
Comparison of certification and training opportunities for each healthcare professional category by country at national and local level

Our aim was to map current training and education opportunities for IPC professionals in Europe and to define local needs for IPC training and highlight differences in training programmes. This should inform stakeholders and enable them to tailor their training programmes to specific needs and to harmonise IPC training in Europe. From July 2018 to February 2019, IPC experts involved in IPC training and education nationally and/or internationally were invited to provide a description of IPC training opportunities and needs in their country. The IPC experts were identified via the EUCIC network and ECDC-related projects on education. In order to reflect the situation across all European regions, eleven countries were selected as indicators and are presented below (Table 1 and Fig. 1).

**Table 1:** Summary of IPC professionals’ profiles, training opportunities and current training needs

	IPC doctor profile and training	IPC nurses profile and training	Other IPC training opportunities	Training Needs
Cyprus	Microbiologist or ID specialist or other doctors No IPC certification No qualification mandated	Work experience in related fields No qualification mandated	Link Nurses: 60-h training programme One 90-ECTS IPC Master program	National training program covering all fields of IPC
England	Clinical Microbiologists or other doctors No qualification mandated	Registered with the nursing and midwifery council No qualification mandated Pending: Advanced nursing practice in IPC, nursing assistant	Master in IPC Short courses by Hospital Infection Society and Royal College of Nursing	Clearer training pathway into IPC specialization including training requirements and assessment
France	Medical doctors (microbiology, PH, ID) or pharmacists No qualification mandated 2017: Cross sectional specialised training for medical internship	Nurses Laboratory technicians No qualification mandated 2019: Advanced nursing practice in IPC	38 post-graduate University degrees in IPC of 1–3 years	Course targeting specific populations and topics Key domains (ie.implementation) should be covered
Germany	Certified medical specialty (48-month training at a facility which has an authorization for postgraduate medical IPC education and 12-month clinical training); final board examination Doctors: 60-month postgraduate training; medical specialty Doctors: IPC training open to all specialists (200 h) as advanced training	Certified nurse specialty (12-month training program)	40-h course to become link doctor or link nurse	Increase training opportunities by creating professorships at every university to ensure (1) IPC training of all medical students (2) junior staff promotion (3) structural integrity of long-term IPC programmes
Greece	Microbiologists, ID specialists or other clinicians with 5-year experience in ID or IPC No qualification mandated	Nursing university degree and 5-year working experience	5-day training seminar by Greek Society for Infection Control	Standardised country-wide training program Include IPC training in the curricula of ID and Microbiology
Italy	Doctors specialized in Hygiene and Preventive Medicine or Infectious diseases No qualification mandated No IPC certification	One official Master course in IPC (since 2014) Otherwise no specific qualification required	Short courses at hospital and regional level or within scientific societies on specific topics Accredited online and on-site courses to become link doctors and link nurses	Standardized methodology and approach to IPC training at national level
Netherlands	Clinical microbiologists 3 months training on IPC out of 60-month specialty training in clinical microbiology	Registered nurses or lab technicians following a training programme of 38 lectures and a minimum of working experience of 18 (Utrecht) to 24 months (Groningen) in an IPC department, as well as writing a thesis	Short courses available for link nurses	Duration of IPC training for doctors too short Lack of expert nurses in IPC
Poland	Medical Doctors who have completed training course or certified clinical microbiologists, epidemiologists, ID, PH or healthcare management specialists IPC training included in specialty training (4 months out of 48-month clinical microbiology and epidemiology training programme, or 3 days in infectious diseases training programme)	Registered nurses with 2 years of work experience following a 2-year training program (844 h) qualification program	Master program in IPC but not official qualification	Create an independent specialization for doctors and determining the legal frameworks for employment Financial incentive to be addressed for nurses
Romania	ID and epidemiology involved in hospital IPC activities	IPC is not regulated as stand-alone nursing specialization Formal training and position, following 3-year post-highschool training, followed by a form of postgraduate training in IPC	CME courses organised each year by different medical universities, on topics related to IPC CNE courses organised each year by the Order of General Medical Nurses, Midwives and Medical Nurses, on topics related to IPC	Standardisation and clarification of recognised training, curricula and tasks for IPC doctors and nurses Improve the consistency of the specialty
Spain	Physicians in preventive medicine and public health (4-year post-graduate period combining 1 year Master degree in public health and clinical residency)	No nationally recognised specialty Employment by public organization require scores on national examinations	Several postgraduate university courses in IPC and AMS or Master degrees (15–60 ECTS) Variety of short courses endorsed by societies	Urgent holistic approach to education and training is warranted Focus on quality improvement, implementation science and communication Align national nurse competencies with European domains
Switzerland	Doctors with 3-year working experience in any field and 3-year working experience in ID and board examination and 1-year working in IPC again with board examination and at least 1 scientific paper published in IPC field	Certified nurse specialty (2-year IPC training programme after basic studies, including final exam and thesis)	6-day training to become a link nurse	Strengthening the importance of needs-appropriate IPC training for implementation of minimum requirements for IPC in hospitals

AMS, antimicrobial stewardship; ECTS, European Credit Transfer and Accumulation System; ID, infectious diseases; IPC, infection prevention and control; PH, public health

## Methods

The current project is an initiative designed and implemented by the EUCIC (namely CT, GB, ET, NTM). EUCIC is a standing committee initiated by the European Society of Clinical Microbiology and Infectious Diseases (ESCMID), to support the global efforts of standardising and harmonizing IPC measures across Europe, to foster research in IPC and to provide training in IPC. EUCIC has one representative from each country, who is a nationally active expert in IPC (background in Microbiology, Infectious Diseases, Public Health) and is member of their national EUCIC committee. The EUCIC network now covers > 90% of all European Union and European Economic Area countries.

A total of 11 countries from the EUCIC network (Cyprus, France, England, Germany, Greece, Italy, Netherlands, Poland, Romania, Spain, Switzerland) were selected by CT, GB and NTM, aiming to include countries in the higher and lower population tier and to cover all EUCIC network geographic regions. IPC experts who were EUCIC members from these countries were personally invited during July 2018–February 2019 and accepted to participate. Following acceptance, they were provided with a prespecified set of points to complete related to IPC education and training in their country; these points addressed:

- Whether IPC is a standalone specialty for healthcare professionals and/or whether there is a national recognition process for IPC training programmes.
- Education and training opportunities in IPC for different healthcare professions (nurses, doctors, pharmacists, other).
- Available training programmes for link nurses. Link nurses represent a link or intermediary between their clinical ward and IPC teams and their role pertains to raise awareness on practical and educational IPC issues and to help in prompt detection of problems such as outbreaks [6].
- Available training programmes (postgraduate, clinical specialty/subspecialty, continuous professional development programmes, short courses, etc.).
- A personal perspective of local needs for improvement of education and training in their country.

After responses were collected, information was compiled and presented by country and healthcare profession.

## Findings

### Cyprus

#### Current educational opportunities

In Cyprus, an IPC training pathway is not clearly defined and not officially certified. For doctors,, no formal IPC training programmes exist currently. Doctors covering IPC-related duties in hospital infection control committees mainly are microbiologists or infectious diseases specialists, although any clinical doctor with work experience in IPC or infectious diseases may be appointed.

One full-time equivalent (FTE) IPC nurse per 250 beds is mandated in public hospitals, with a required background (training or work experience) in the field of IPC, infectious diseases or public health. A financial support from the Ministry of Health and the Cyprus Nurses and Midwives Association is provided to most IPC nurses to train at seminars organised by the Greek Society of Infection Control. In 2013, the Nursing Services of the Ministry of Health opened a 60-h training programme dedicated for link nurses in all public hospitals of Cyprus [7]. In September 2018, the School of Medicine, European University Cyprus, initiated a 90-ECTS Master programme in the prevention and control of infectious diseases for healthcare professionals.

#### Local needs

Although a country-wide educational programme is running on an annual or biannual basis, it is only aimed at nurses, especially link nurses. The limited national resources allocated to training of IPC professionals necessitate the establishment of a national training programme covering all fields of IPC [5].

### England

#### Current educational opportunities

Several pathways exist to become an IPC professional in England, despite no required ‘set’ or baseline qualification. A director of infection prevention and control (DIPC), appointed on each healthcare organisation, is responsible for the overall performance of IPC activities.

Most IPC doctors are also microbiologists as IPC and microbiology are closely aligned.

Nursing practice in IPC does not require any mandated, additional qualifications. Whilst it is possible to practice in IPC without specialist postgraduate qualifications, these become essential to gain promotion to more senior,advanced clinical, or managerial roles.

Various optional courses and modules are available, aiming to improve IPC knowledge and practice, at Master or PhD level. These courses are undertaken depending on individual need, funding and availability. For example, a Master in IPC is delivered online, affording flexibility to students to continue working full-time [8]. Dependent on funding, continuing professional development (CPD) courses may be offered to IPC specialists by their organisation. Learned societies such as the Hospital Infection Society (HIS) also provide short courses [9], including the “Foundation Course on Healthcare Infection Control”, a 3-day, non-residential, stand-alone course held annually. In 2017, the HIS launched the DIPC Network and Development Programme directed at DIPCs. More specialised, a course on Engineering Aspects of IPC explores hospital technological components (i.e. operation room ventilation, endoscopy, sterilization). Finally, the Royal College of Nursing recently launched an IPC module to enable the development of clinical and leadership skills in this area [10].

#### Local needs

Although some competencies for practitioners in IPC have been published [11], a clearer training pathway into IPC specialization (including training requirements and assessment) would be extremely beneficial for nurses interested in developing and progressing their career within IPC. Two fairly radical workforce changes have to be highlighted. The forthcoming recognition of advanced nursing practice (where nurses embrace roles and tasks traditionally performed by other professionals) and the introduction of nursing associates—bridging the care and skills of nurses and nursing assistants—would demand that IPC training opportunities consider the panoply of roles involved in optimal clinical and organisational practice [12, 13].

### France

#### Current educational opportunities

In France, IPC is an overspecialization with a community consisting of healthcare professionals of various backgrounds (clinical and non-clinical). This includes medical doctors (mainly microbiologists, public health specialists, infectious diseases specialists), nurses with various backgrounds, pharmacists (specialised or not in microbiology) and laboratory technicians. Hospitals are mandated to appoint one FTE IPC practitioner and nurse per 800 and 400 beds, respectively.

The most common way to become an IPC specialist is through a postgraduate university degree. In 2010, this degree was available in 38 different universities to all healthcare professionals with a prerequisite of an undergraduate degree (3 years of university education after the baccalauréat). Most of them are based on a part time one year course (range 1–3 years) amounting to 100–200 teaching hours (range: 70–420 h), approaching the same topics (healthcare-associated infections, epidemiology, microbiology, infection prevention, quality and safety, sterilization, environment, management). Students in these courses are usually in post in IPC teams or in clinical/non-clinical wards, in permanent positions or in training (interns). At the end of the year, a final exam is organised, with a thesis to defend for some university degrees. Every year, around 400 students graduate in France.

In April 2017, a framework was developed to formalise the educational pathway of IPC specialization during medical studies, in three stages: basic, in-depth and consolidation. This option titled “cross sectional specialised training” is planned to be open to several medical internships: public health, pathology and infectious diseases. The number of students will be regulated annually by the Ministry of Health. A mandatory system of CPD is in place for practitioners and nurses, which is also applicable to IPC specialists. The IPC specialist curriculum and the advanced nursing practice roles in IPC have been recently defined by the French society for IPC [14].

#### Local needs

In France, IPC education programmes are highly heterogeneous in terms of targeted populations, contents and methods. Courses usually involve a large spectrum of professionals, from different backgrounds, engaged in courses for various educational purposes. Usual criticisms on IPC French educational programmes are: the validation process, the variability of topics approached, and the lack of follow-up once the degree is obtained. Developing courses targeting specific populations, addressing specific topics and more based on practices rather than general knowledge, would be a positive evolution of the current schemes. Key domains like implementation science, need to be considered as a priority.

### Germany

#### Current educational opportunities

In Germany, IPC is a certified medical specialty (*Facharzt für Krankenhaushygiene und Umweltmedizin*). As in other medical specialties, a 60-month postgraduate training must be completed after medical school to become an IPC specialist. This training is split in 12 months spent in clinical wards (i.e., internal medicine, surgery, pediatrics etc.) and 48 months in a certified and authorised IPC department. During the latter period, trainees can spend 12-month rotation in a microbiology department. The specialty of clinical microbiology is distinct to IPC in Germany. Clinical microbiologists are allowed to work in IPC with the main restriction to not participate as trainers in IPC training programmes. The German Medical Chamber is responsible for the training curriculum and for providing educational permission to certified IPC specialists [15]. The training programme covers a large panel of topics (i.e., outbreak management, epidemiology, diagnostic and laboratory techniques, technical hygiene, waste, and air and water management). After completion of this 60-month period, trainees undergo an oral examination to complete their training and graduate. In 2010, a new kind of structured IPC training, open to all medical specialists, was announced by the German Medical Chamber to fill the lack of IPC resources. This so called “structured training in infection control” consists of a 200-h course in which different modules cover basic aspects of IPC. If only the first module (40 h) is completed candidates can become link doctors (*Hygienebeauftragte Ärzte*). By completion of the whole 200-h course and additional working experience in IPC of usually 24 months, candidates can act as an IPC specialist. However, compared to the actual medical IPC specialists, they do not have the right to train others. Hence, this implies consequences for the promotion and training of future IPC generations.

Nurses in Germany undergo a 12-month (full-time, 24-month part-time) training programme, including a final exam and a thesis, to become state-approved and professionally recognised IPC nurses (*Hygienefachkraft*). Registered nurses with at least two years of working experience after graduating are eligible for this training. Furthermore, a 40-h IPC course is available for all nurses to become link nurses (*Hygienebeauftragte Pflegekräfte*).

#### Local needs

Germany has a long tradition in IPC, however, it represents a rather small specialty in comparison to others, lacking professorships or independent IPC sections in some university hospitals and in many other larger hospitals. Creating IPC professorships at every university would ensure (1) IPC training of all medical students (2) junior staff promotion, as well as decreasing staff shortage and (3) structural integrity of long-term IPC programmes.

### Greece

#### Current educational opportunities

In 2014, a ministerial decision mandated the creation of IPC committees in all hospitals, that includes a full-time IPC nurse (ratio of 1 post per 250 beds) [16].

Training in IPC is not mandatory for medical doctors of any specialty. In most hospitals, microbiologists, infectious diseases specialists, or other clinicians with work experience in the field of infectious diseases or IPC, can be members of the IPC committees. According to the same decision, the IPC nurse, the infectious diseases doctor and the microbiologist, comprise the so-called intervention team of the IPC committee.

A nursing university degree and at least five years of clinical experience are required to be appointed as an IPC nurse. Work experience in an IPC team, as a link nurse or postgraduate training in infectious diseases, IPC, public health, epidemiology, public administration or intensive care, are considered additional criteria for their selection.

A 5-day training seminar (approximately 45 h), endorsed by the Ministry of Health and the Hellenic Center for Disease Control and Prevention, is organised annually by the Greek Society for Infection Control and addresses all healthcare professionals regardless of background. This seminar includes interactive lectures and workshops on various topics in IPC.

Some Master programmes in Nursing and Medical Schools include courses on IPC in their programme.

#### Local needs

A standardised country-wide training programme in IPC is needed in Greece to meet the mandatory needs of IPC nurses and committees in all hospitals. The content of such an educational programme, either in the form of a sub-specialization or continuous professional development programme, should be compatible with published competencies for IPC professionals. Finally, mandatory inclusion of antimicrobial stewardship (AMS) and infection control training in the curricula of the specialties of infectious diseases and microbiology should be considered.

### Italy

#### Current educational opportunities

In Italy, IPC has historically been part of the broader field of Hospital Hygiene mandated for medical doctors specialized in Hygiene and Preventive Medicine. There is not a specific specialization in IPC but during the four years postgraduate training in Hygiene, doctors attend several courses on hospital and environmental hygiene, epidemiology and prevention of infectious diseases, surveillance, clinical microbiology, research methodology and statistics, communication and information to the public. Training comprises hospital and community internships of variable duration depending on the organization of each of the 34 Italian postgraduate schools of Hygiene and Preventive Medicine.

Regarding nurses, it is mandatory for hospitals to establish an infection control committee and to provide at least one FTE nurse dedicated to hospital infection control per 250 beds. Some national societies and associations have made several attempts to measure the educational needs and to build a national curriculum for nurses to face the issue of hospital acquired infections. Several courses have been initiated mainly for nurses, even though not continuously, neither in a uniform way among the 20 Italian regions, besides short courses and educational initiatives at hospital and regional level. At present, since 2014, there is an official Master course for nurses organized by the nurse association for the prevention and control of infectious risk (ANIPIO), that lasts one year and can be obtained currently in four Italian universities. The program consists of interactive lessons, simulations, role-playing, tutored internships in hospital, project works and a final exam. The mastered nurse is intended to work within the hospital committee dedicated to infection control and antimicrobial stewardship, coordinated by a hygienist (medical doctor specialized in Hygiene and Preventive medicine) and composed by at least an infectious diseases physician, a microbiologist, a pharmacist and representatives of the medical and surgical departments.

#### Local needs

Despite numerous attempts to provide a national standard of education, there is huge heterogeneity among Italian regions regarding the competencies and skills required of an IPC professional in nurses and medical doctors. During the Hygiene and Preventive Medicine school, the medical doctor risks to not receive an appropriate education regarding specific aspects of IPC due to the many other topics needed to be covered during the postgraduate training. The few Master courses available for nurses provide only a small number of specialized professionals in respect to the country needs.

### Netherlands

#### Current educational opportunities

In the Netherlands, IPC is an integral part of training in clinical microbiology (*Specialist Medische Microbiologie*). Specialty training in clinical microbiology comprises of a 60-month post-graduate training programme for medical doctors, consisting of multiple rotations [17]. The main rotations are laboratory training, IPC (3 months), consultation and interdisciplinary visits, public health, scientific research, and laboratory management. Interim self-evaluation tests are performed without a formal final examination and not impacting the training progress.

In addition to training for medical doctors, a certified position named “expert nurses in infection prevention” (*deskundige infectiepreventie*) consists of training in only two Universities in the Netherlands (Utrecht and Groningen). The programme includes 38 lectures and a minimum of 18 (Utrecht) to 24 months (Groningen) work in an IPC department is required to complete the training, as well as a thesis. Candidates with at least a higher educational institute qualification are eligible for the training programme.

Hospitals and private institutions provide short post-graduate courses for link nurses. Doctors do not need to follow any course or receive any particular IPC training to become an IPC link in clinical wards.

#### Local needs

Although IPC is an integral part of the training programme in the specialty of clinical microbiology, its duration is not optimal, since only three out of the 60 months are solely dedicated to IPC. Additionally, probably due to the limited training opportunities (only two centers in the whole country), “expert nurses in infection prevention” are lacking in the Netherlands. Additional training opportunities for both doctors and nurses would be desirable.

### Poland

#### Current educational opportunities

According to current law in Poland, a doctor in charge of hospital IPC has to be a specialist either in epidemiology, clinical microbiology, infectious diseases, public health, or healthcare management/organisation [18, 19].

IPC is included in the 48-month clinical microbiology and epidemiology training programme as 4 months reserved for IPC, but it is poorly represented in infectious diseases training programmes as a 3-day course. Any doctor completing a relevant qualification course (1- or 2- week training programme) can lead a hospital IPC programme.

For two decades, IPC specialization training for nurses and midwifes (so-called “epidemiological nursing”) is available in Poland. For successful completion a nurse or midwife has to follow a dedicated two-year training programme (currently 844 h). Only registered nurses with at least two years of working experience are eligible for the programme. One FTE IPC nurse per 200 beds is required in hospitals.

The Jagiellonian University Medical College in Cracow offers a Master programme in IPC for healthcare professionals, i.e. doctors, nurses and other B.Sc holders. However, completing this programme is not officially identified as a required qualification for IPC team members.

#### Local needs

For doctors, qualifications and skills are highly inconsistent due to varying backgrounds and training. The main local need would be first to create an independent specialization for doctors (i.e. infection control and hospital epidemiology) and determining legal frameworks for employment of the doctors (number of beds per FTE and form of employment). In the case of nurses, the vast majority of Polish hospitals employ IPC nurses as mandated by the ministry of health. However, appointed nurses are not always fully dedicated to IPC, mainly due to lack of financial incentives, and particularly as one FTE per 200 beds seems to be too low for large, tertiary hospitals in the era of emerging antimicrobial resistance (AMR). In addition, financial incentives should also be addressed.

### Romania

#### Current educational opportunities

In Romania, IPC is not regulated as stand-alone medical or nursing specialty. Hospital IPC activities are shared by infectious diseases specialists and epidemiologists, and by each chief of a clinical ward. Since 2016, national legislation specifically regulates the existence of one IPC department per hospital, directly subordinated to hospital management [20]. The department includes at least one epidemiologist per 400 beds (the medical specialty of epidemiology is obtained following a post-graduate residency training of 4 years), one person designated as being responsible for AMS per 400 beds (either an infectious diseases specialist—specialty obtained after residency training of 5 years, or another clinical doctor trained in IPC or AMS), and one IPC nurse per 250 beds.

Most of the medical schools in Romania run continuing medical education (CME) courses related to IPC topics each year. Topics included in the programme are antimicrobial prophylaxis and treatment, epidemiology and surveillance, IPC and microbiology. CME courses range from 4 to 40 h of theoretical and practical training.

To be appointed in IPC teams, nurses need to complete at least a 3-year post-high school training as general medical nurse (which includes 42 h of training in microbiology, virology and parasitology, and 44 h of training in hygiene and IPC), along with a form of postgraduate training in IPC. This postgraduate training can either be an accredited specialization of hygiene and public health nurse obtained after a 2-year training organized by the National School of Public Health and ending with a practical and written exam or another type of accredited continuing nursing education (CNE) course on IPC-related topics. CNE courses generally have a duration of 2–5 days and are organised periodically, either online or onsite, in most counties by the Order of General Medical Nurses, Midwives and Medical Nurses. Training courses are not standardised at the national level in terms of types, duration, and content.

#### Local needs

IPC is not officially regulated as a medical or nursing specialty in Romania, with other connected specialties sharing responsibility for hospital IPC activities. Following the introduction in 2016 of the national regulation that each hospital should have an IPC department, and that this department should include at least one epidemiologist, one AMS doctor, and a nurse, a degree of standardization has been obtained. However, heterogeneity still exists among different training programmes for both doctors and nurses. A standardization and clarification of curricula and tasks devoted to IPC and AMS doctors and nurses would improve consistency.

### Spain

#### Current educational opportunities

In Spain, typical IPC activities are the responsibility of doctors in Preventive Medicine and Public Health. These medical specialists undergo a 4-year post-graduate training combining an initial 1-year Master degree in Public Health with clinical residencies in various domains related to public health [21]. In 2018, 65 training posts in this specialty were opened in the country. In relation to IPC activities and training, residents spend a 9-month rotation at a Preventive Medicine service, where they achieve proficiency in surveillance and control of infections. A sustained and vigorous grass root movement amongst physicians working in infectious diseases lobbied successive national governments to approve a national specialty in that area.

Regarding nurses, IPC is equally not included in the portfolio of nationally recognized residency-based postgraduate training. In 2018, the national regulatory body for nurses published a resolution detailing the competencies and sphere of practice in IPC, including health promotion and disease prevention [21]. At the time, the only other institutional document published was the proposal of nursing competencies released by the Spanish Association of Nurses in Infection Prevention and Control [22]. Up until now (August 2020), however, there has been no further national or local progress in terms of development or implementation of either set of competencies.

Other educational opportunities available for healthcare professionals include several postgraduate university courses in IPC and AMS at diploma or Master’s level, varying from 15 to 60 ECTS which are delivered either online or via conventional face-to-face sessions. A variety of short courses and training days are endorsed by the Spanish Society of Preventive Medicine and Public Health [23] and/or the Spanish Society of Infectious Diseases and Clinical Microbiology [24].

#### Local needs

Whilst there are successful initiatives in IPC, an urgent, holistic approach for education and training in these areas is warranted. The existing learning opportunities are largely offered separately to each professional group. Additionally, the growing attention demanded by IPC practice in terms of quality improvement skills, implementation science, and communication, should direct course organisers to develop these fields in course curricula. Regarding nurses, it is not yet clear how the current national competencies align with the domains of practice included in recent European core documents [3]. Finally, the scoring process for employment in the state-funded national health service, in addition to registration fees, are barriers for the adoption of non-nationally recognised external qualifications.

### Switzerland

#### Current educational opportunities

After January 2021, IPC is recognised as a federally regulated subspecialty for doctors holding a subspeciality title in infectious diseases.The requirements for infectious diseases are a minimum of 3 years working experience in internal medicine, plus 3 years in infectious diseases, including a board exam. After passing the exam, the infectious diseases doctor trains for 1 year in a recognized center for IPC, again with an exam and a published scientific paper on IPC. Post-doc training for physicians is organised by *Schweizerisches Institut für ärztliche Weiter- und Fortbildung* (SIWF) [25].

In Switzerland, IPC training for nurses is federally regulated since 2009 and all IPC nurses must pass a final national exam. Nurses with a diploma in nursing (*dipl. Pflegefachfrau*) or a B.Sc. in obstetrics, 2 years of professional experience and at least 1 year of professional practice in infection prevention, undergo a 2-year training programme, including a national final exam and a thesis, to become a state-approved IPC nurse (*Fachexperte/-in für Infektionsprävention im Gesundheitswesen mit Eidgenössischem Diplom*) [26].

Nurses can also participate in a 6-day training to become link nurses. The training plan to become a link nurse was developed in cooperation with the Swiss Society for Hospital Hygiene (SGSH) and the experts Infection Prevention & Consultants Hospital Hygiene (*Fachexperten/-innen Infektionsprävention & Berater/-innen Spitalhygiene* fibs). Requirements for the training are a diploma in nursing (*dipl. Pflegefachfrau/ Pflegefachmann*) or a B.Sc. in obstetrics and at least 1 year of professional experience after graduation [27]. National minimum requirements have been developed and are in the process of evaluation by the authorities, as by the medical board.

#### Local needs

For the Federal Council in Switzerland, reduction of healthcare-associated infections is a top priority. It approved a National Strategy for the Monitoring, Prevention and Control of Healthcare-Associated Infections (NOSO strategy) a priority measure in the overall health policy review "Health 2020". One of the key actions is strengthening the importance of infection prevention in education and training [28], aiming to promote awareness on the importance of infection prevention in healthcare institutions and reinforce continuing professional development. For this, a needs-appropriate training will require a pool of facilitators (doctors, nurses or link nurses), trained as specialists in infection prevention.

## Discussion

The current report highlights the variability in content, duration, recognition (such as equivalences and professional or government body requirements), and assessment (such as evaluation, thesis) of IPC specialists’ education and training across European countries. IPC specialty is nationally recognized in few countries, namely France, Germany, Switzerland for both doctors and nurses, and in Poland and the Netherlands only for nurses. Furthermore, the different background of IPC specialists in some countries including clinical doctors, microbiologists, pharmacists, nurses and technicians, requires adjustment of the training programmes to defined learning outcomes and competencies [3, 11], to bridge the gap in IPC knowledge and skills between different professionals. Educational strategies employed by countries also appear heterogeneous, with some strict and intensive schemes (e.g. Netherlands for nurses, Germany for doctors and nurses) and more flexible formats (e.g., Greece and Cyprus). A standardization of topics, competencies and volume may improve the educational process and harmonise the educational outcomes not only within, but also between European countries. Among local needs, a clear education pathway based on defined IPC competencies, associated with an official recognition by national or international health authorities of IPC as a specialty, might increase human resources and education and training opportunities in this field. This is especially critical and warranted for countries facing high rates of healthcare-associated infections and AMR.

In 2014, infection control qualifications were board-certified in only 17% of European countries, whereas more than a third of countries had no national curriculum or training programme for doctors or nurses [29]. In addition, in most countries, training programmes specifically in IPC for other healthcare professionals, such as pharmacists, are either absent, or of limited extent (i.e., short-term postgraduate seminars). As IPC is an interdisciplinary field that requires active contribution and shared knowledge between healthcare professionals of different backgrounds, training and staffing needs in IPC should be directed towards healthcare professionals of all backgrounds. This has become even more evident during the current coronavirus disease 2019 (COVID-19) pandemic, where national and international guidelines highlight the need to educate and train all healthcare professionals on IPC [30–32].

There are some important initiatives aiming for harmonisation of education and training in IPC [5]. For example, the EUCIC has implemented the “European Training Programme in Infection Prevention and Control”, a 2-year programme intended for healthcare professionals of different backgrounds [33]. The programme aims to cover all essential competencies required to become an IPC professional [3, 5], but also to address local needs and build capacity. Various short-duration educational modules are included, with mandatory basic and advanced modules, as well as local modules organised in each European country, addressing local issues. The programme is organised in collaboration with the ECDC. Furthermore, following a formal proposal, a Multidisciplinary Joint Committee on Infection Control (MJC IC) was created in 2018 in the European Union of Medical Specialists (UEMS). The scope of the MJC IC is education of European medical specialists in IPC, aiming to define European standards of medical education and training in IPC and to incorporate IPC core competencies in the curricula of relevant medical specialties.

We should acknowledge certain limitations in regard to the present article. Firstly, the sample selected for description was purposeful and not exhaustive. Secondly, although guidance to search for information was given, there was no congruent search strategy and in several cases, information was based on personal knowledge and personal communications. However, to apply a unified search strategy for all countries would not have been possible due to the heterogeneity of information and sources.

## Conclusions

This report provides a detailed picture of the current situation and the needs to improve IPC training in different countries representative of all European geographic regions. Training opportunities for each healthcare profession were sought, as well as national training programmes, university curricula and link nurse training, in order to produce detailed descriptions and to facilitate meaningful comparisons. The observed heterogeneity in IPC training between European countries may be eliminated through establishment of interdisciplinary region-wide training programmes, with common learning objectives, shared know-how and support by national and international professional bodies.

## Data Availability

Data sharing is not applicable to this article as no datasets were generated or analysed during the current study.

## Abbreviations

AMR: Antimicrobial resistance
AMS: Antimicrobial stewardship
B.Sc: Bachelor of Science
CME: Continuing medical education
CNE: Continuing nursing education
CPD: Continuing professional development
DIPC: Director of infection prevention and control
ECDC: European Center for Disease Prevention and Control
ESCMID: European Society of Clinical Microbiology and Infectious Diseases
EUCIC: European Committee of Infection Control
FTE: Full-time equivalent
HIS: Hospital Infection Society
IPC: Infection prevention and control
MJC IC: Multidisciplinary Joint Committee on Infection Control
PROHIBIT: Prevention of Hospital Infections by Intervention and Training study
SGSH: Swiss Society for Hospital Hygiene
SIWF: Schweizerisches Institut für ärztliche Weiter- und Fortbildung
TRICE: Training in Infection Control in Europe
UEMS: European Union of Medical Specialists
